# Aryl Hydrocarbon Receptor Downregulates MYCN Expression and Promotes Cell Differentiation of Neuroblastoma

**DOI:** 10.1371/journal.pone.0088795

**Published:** 2014-02-21

**Authors:** Pei-Yi Wu, Yung-Feng Liao, Hsueh-Fen Juan, Hsuan-Cheng Huang, Bo-Jeng Wang, Yen-Lin Lu, I-Shing Yu, Yu-Yin Shih, Yung-Ming Jeng, Wen-Ming Hsu, Hsinyu Lee

**Affiliations:** 1 Department of Life Science, National Taiwan University, Taipei, Taiwan; 2 Institute of Cellular and Organismic Biology, Academia Sinica, Taipei, Taiwan; 3 Institute of Biomedical Informatics, National Yang-Ming University, Taipei, Taiwan; 4 Degree Program of Translational Medicine, Academia Sinica–National Taiwan University, Taipei, Taiwan; 5 Department of Clinical Laboratory Sciences and Medical Biotechnology, College of Medicine, National Taiwan University, Taipei, Taiwan; 6 Department of Pathology, National Taiwan University Hospital and National Taiwan University College of Medicine, Taipei, Taiwan; 7 Department of Surgery, National Taiwan University Hospital and National Taiwan University College of Medicine, Taipei, Taiwan; McGill University, Canada

## Abstract

Neuroblastoma (NB) is the most common malignant disease of infancy. MYCN amplification is a prognostic factor for NB and is a sign of highly malignant disease and poor patient prognosis. In this study, we aimed to investigate novel MYCN-related genes and assess how they affect NB cell behavior. The different gene expression found in 10 MYCN amplification NB tumors and 10 tumors with normal MYCN copy number were analyzed using tissue oligonucleotide microarrays. Ingenuity Pathway Analysis was subsequently performed to identify the potential genes involved in MYCN regulation pathways. Aryl hydrocarbon receptor (AHR), a receptor for dioxin-like compounds, was found to be inversely correlated with MYCN expression in NB tissues. This correlation was confirmed in a further 14 human NB samples. Moreover, AHR expression in NB tumors was found to correlate highly with histological grade of differentiation. *In vitro* studies revealed that AHR overexpression in NB cells induced spontaneous cell differentiation. In addition, it was found that ectopic expression of AHR suppressed MYCN promoter activity resulting in downregulation of MYCN expression. The suppression effect of AHR on the transcription of MYCN was compensated for by E2F1 overexpression, indicating that E2F1 is involved in the AHR-regulating MYCN pathway. Furthermore, AHR shRNA promotes the expression of E2F1 and MYCN in NB cells. These findings suggest that AHR is one of the upstream regulators of MYCN. Through the modulation of E2F1, AHR regulates MYCN gene expression, which may in turn affect NB differentiation.

## Introduction

Neuroblastoma (NB) is a childhood tumor derived from a sympathoadrenal lineage of neural crest progenitor cells, and is the most common malignant disease of infancy. NB cells exhibit similar characteristics to undifferentiated cells and often metastasize to distant organs [1]. Approximately 60% of patients diagnosed with NB display a stage IV disease and have very poor prognosis. The 5-year survival rate of patients with NB is no more than 30%, even with aggressive therapy [2]. As a result, 50% of patients with NB die from this tumor.

The clinical presentation of NB can be categorized into three distinct patterns: (i) life-threatening progression; (ii) maturation to ganglioneuroblastoma (GNB) or ganglioneuroma (GN); and (iii) spontaneous regression [3]. Recent evidence suggests that NB cells exhibit the capacity to differentiate into mature cells and can be forced to differentiate upon treatment with retinoic acid, butyric acid, or cisplatin [4], [5]. A number of molecules normally expressed during embryonic development, including HNK-1, neuropeptide Y, tyrosine hydroxylase, TrkA, and CD44, are found in NB tumors [6], [7], suggesting that the tumorigenesis of NB could be a divergence of the embryonic development of the sympathetic system. On the other hand, NB cells with better prognosis are often found to express markers indicative of cell differentiation, such as HNK-1 or TrkA [8], [9]. Therefore, it is plausible that the tumorigenesis of NB may result from defective differentiation of embryonic NB cells [5]. However, what factors may contribute to the regulation of NB cell differentiation is still unclear.

MYCN is one of the most well-known prognostic markers of NB [10]. Amplification of MYCN occurs in approximately 25% of all and approximately 40% of advanced NB cases [11]. MYCN belongs to the Myc family of basic-helix-loop-helix transcription factors that play a critical role in regulating cell proliferation, differentiation, apoptosis, and oncogenesis. Patients with NB with tumors containing a single copy of MYCN usually have a favorable prognosis, whereas amplification and/or MYCN overexpression are closely associated with rapid disease progression and a high mortality rate [12]. The expression level of MYCN has also been shown to be correlated with the activation of genes associated with tumor aggression [13]. Furthermore, MYCN expression in non-amplified NB cell lines can induce quiescent cells to re-enter into the cell cycle [14]. Transgenic mice with neuroectodermal-specific expression of MYCN may develop NB spontaneously [14]. These lines of evidence suggest that MYCN has a great effect on NB cell behavior. However, how MYCN expression is regulated in NB remains unknown.

In this study, we employed an oligonucleotide microarray and bioinformatics approach to evaluate the genes associated with MYCN expression in NB. Aryl hydrocarbon receptor (AHR) was found to be inversely correlated to MYCN expression. AHR is an 805-amino acid-long ligand-activated transcription factor that possesses a basic-helix-loop-helix (bHLH)/Per-Arnt-Sim domain and mediates the biological effects of dioxin-like compounds [15], [16]. In addition to its roles as a toxicity mediator, AHR has also been shown to be an important regulator of cell death, proliferation, differentiation, and cell cycle progression [17]–[20]. Furthermore, AHR overexpression has been shown to induce neural differentiation of NB cells [21]. In addition, dioxins may inhibit NB cell proliferation via AHR-mediated G1 arrest [22]. AHR is also critical for the regulation of neuronal development in *Caenorhabditis elegans* [23]. These findings strongly suggest that AHR may play an important role in the tumorigenesis of NB. Thus, the aim of this study was to investigate the relationship between AHR and MYCN in NB.

## Materials and Methods

### Ethics Statement and Patients Tissues

The use of human tissues for this study was approved by the National Taiwan University Hospital Research Ethics Committee. Written informed consent was obtained from patients before samples were collected. Tumor samples were obtained during surgery and immediately frozen in liquid nitrogen. The categorization of tumor histology was based on the International Neuroblastoma Pathology Classification scheme [24]. MYCN status was determined by fluorescence *in situ* hybridization analysis of formalin-fixed paraffin-embedded tissues or fresh tumor single cells [25], [26].

### Tissue Oligonucleotide Microarray Analysis

Purified RNAs were quantified by OD260nm using an ND-1000 spectrophotometer (Nanodrop Technology) and RNA quality was checked by Bioanalyzer 2100 (Agilent Technology). Total RNA (0.5 µg) was amplified by a low RNA input fluor linear amp kit (Agilent Technologies, USA), and labeled with Cy3 or Cy5 (CyDye, PerkinElmer) during the *in vitro* transcription process. Tumor RNA was labeled by Cy5 and RNA from Universal Human Reference RNA was labeled by Cy3. Furthermore, 2 µg of Cy-labeled cRNA was fragmented to an average size of approximately 50–100 nucleotides by incubation with a fragmentation buffer at 60°C for 30 min. Correspondingly fragmented labeled cRNA was then pooled and hybridized to Human 1A (version 2) oligo microarray (Agilent Technologies) at 60°C for 17 h. After washing and drying by nitrogen gun blowing, microarrays were scanned with an Agilent microarray scanner (Agilent Technologies) at 535 nm for Cy3 and 625 nm for Cy5. Scanned images were analyzed using Feature extraction 8.1 software (Agilent Technologies), image analysis and normalization software was used to quantify signal and background intensity for each feature, substantially normalizing the data by rank-consistency-filtering LOWESS method [27]. The array data discussed in this publication have been deposited in NCBI's Gene Expression Omnibus (GEO) and are accessible through GEO Series accession number GSE53371.

(http://www.ncbi.nlm.nih.gov/geo/query/acc.cgi?acc=GSE53371).

### Significant Functional Networks and Biological Pathway Annotations

The differentially expressed genes were annotated by Ingenuity Pathway Analysis (IPA; Ingenuity Systems) [28], [29]. In brief, the identified genes were mapped onto available functional networks and specific biological pathways, and then ranked by score. The score was based on a p-value calculation; for example, if the score is 3, then the corresponding p-value is 10^−3^, meaning there is a 1/1000 chance that the focus genes are in a network due to random chance. The significance of the association between the dataset and the canonical pathway was measured in two ways: ratio and p-value. Ratio is displayed as the number of genes from the dataset that map to the pathway divided by the total number of genes that map to the canonical pathway. Fischer’s exact test was used to calculate a p-value determining the probability that the association between the genes in the dataset and the canonical pathway is explained by chance alone. In the statistical analysis, biofunctions and disturbed pathways were sorted in order of significance by using IPA web tool.

### Authentication of Cell Lines

The human NB cell lines SH-SY5Y (ATCCH CRL-2266TM, MYCN norml), SK-N-SH (ATCCH HTB-11TM, MYCN norml), SK-N-DZ (ATCCH CRL-2149TM, MYCN amplified), and SK-N-BE(2) (ATCCH CRL- 2271TM, MYCN amplified) were obtained from the American Type Culture Collection (Manassas, VA, USA) in November 2007. The human NB cell line IMR-32 (BCRC 60014, MYCN amplified) was obtained from the Biosource Collection and Research Center (Hsinchu, Taiwan) in November 2007. Tet21N [A subclone of SH-EP cells expressing *MYCN* from a tetracycline-regulated promoter (Tet-off)] [30] were kindly provided by Dr. Manfred Schwab of the German Cancer Research Center, in January 2009. Care has been taken to maintain the purity of the cultures.

### Plasmid Constructions, Transfection, and Selection of Stable Cell Lines

Human *AHR* was isolated from the human hepatoma cell line HepG2 and cloned into a pEGFP-C1 vector. The newly constructed plasmid was named hAHR-pEGFP-c1. For AHR stable cell line selection, hAHR-pEGFP-c1 was transfected into cells by Lipofectamine 2000 (Invitrogen). Forty-eight hours after transfection, cells were trypsinized from 6-well plates and plated in 10-cm dishes with culture medium (DMEM/F12) containing 600 µg/ml G418 for hAHR-pEGFP-c1 stable clone selection. Media with antibiotics were replaced every two to three days. Approximately one month later, antibiotic-resistant single clones were generated and amplified. Recombinant AHR expression was confirmed by real-time PCR.

### RNA Interference (RNAi) and Lentiviral Infection

shRNA targeted toward AHR and luciferase (pLKO.1-shLuc), a negative control, was obtained from the RNAi core laboratory, Academia Sinica, Taiwan. For the virus package, 293T cells were co-transfected with 7.5 µg pLKO.1 lentiviral vectors, 1.9 µg of envelope plasmid pMD.G, and 5.6 µg of packaging plasmid pCMVΔR8.91. Virus solutions were collected 24 and 48 h after transfection and were condensed using PEG8000. To knockdown the AHR, SK-N-SH cells were treated with virus solution for 24 h. Forty-eight hours after virus transduction, total cellular RNA was extracted and subjected to reverse-transcription.

### Reverse-transcription

Total cellular RNA was extracted from the cells using the TRIzol reagent (Invitrogen). The reverse transcription of 1 µg isolated total RNA was performed in a 20 µl reaction mixture using M-MuLV Reverse Transcriptase (Thermo) and an oligo-dT primer.

### Quantitative Real-time PCR

Real-time PCR reactions were conducted in an iCycler iQ Real-Time detection system (Bio-Rad) using SYBR Green I (ABgene). The thermal profile for PCR was 95°C for 3 min, followed by 40 cycles of 95°C for 30 s, and 60°C for 30 s. Thermocycling was performed with a final volume of 15 µl containing 1 µl of cDNA sample. The melting curve of each tube was examined to confirm a single peak appearance. The sequences of paired primers for real-time PCR detection are as follows: GAPDH forward 5′-GGT GGT CTC CTC TGA CTT CAA C-3′, GAPDH reverse 5′-TCT CTC TTC CTC TTG TGT TCT TG-3′; AHR forward 5′-CTG ACG CTG AGC CTA AGA AC-3′, AHR reverse 5′-ACC TAC GCC AGT CGC AAG-3′; MYCN forward 5′-GTC ACC ACA TTC ACC ATC AC-3′, MYCN reverse 5′-GGG AAG GCA TCG TTT GAG-3′; GAP43 forward 5′-TCC GTG GAC ACA TAA CAA GG-3′, GAP43 reverse 5′-CAG TAG TGG TGC CTT CTC C-3′; NSE forward 5′- TGT CTG CTG CTC AAG GTC AA-3′, NSE reverse 5′-CGA TGA CTC ACC ATG ACC C-3′; CRT forward 5′-GTC GAT GTT CTG CTA TGT TTC-3′, CRT reverse 5′-AAG TTC TAC GGT GAC GAG GAG-3′; Nestin forward 5′-GCC CTG ACC ACT CCA GTT TA-3′, Nestin reverse 5′-GGA GTC CTG GAT TTC CTT CC-3′; Vimentin forward 5′-GAA GAG GTT AGT GGA GTG A-3′, Vimentin reverse: 5′-TGC TGT TCC TGA ATC TGA-3′.

### Western Blot

Proteins were extracted from cell lysates. Cells were lysed in a lysis buffer (25 mM Tris, pH 7.4, 150 mM NaCl, 1% NP-40, 1 mM Na_3_VO_4_, 1 mM PMSF, and 1 g/ml) for 15 min at 4°C. Lysates were spun at 13,000 rpm for 15 min at 4°C, and the supernatant was used for Western blotting. Protein concentration was determined using a Bio-Rad protein assay kit. Concentration-normalized lysates were boiled at 100°C in an SDS sample buffer for 10 min. Proteins were fractionated by SDS-PAGE (150 volts for 1.5 h) and transferred to nitrocellulose membranes (80 Volts for 90 min). Membranes were blocked with 5% BSA in TBS-T (0.1% Tween 20 in TBS), followed by overnight incubation at 4°C with appropriate dilutions of primary antibody in TBS-T. After three washes with TBS-T, membranes were incubated with the appropriate secondary antibody coupled with horseradish peroxidase, and immunocomplexes were visualized using an enhanced chemiluminescence (ECL) kit according to the manufacturer’s instructions. The antibodies used were as follows: rabbit polyclonal anti-AHR antibody (Enzo Life Science, NY, USA); mouse monoclonal anti-MYCN antibody (Santa Cruz, CA, USA); and goat polyclonal anti-β-actin (Santa Cruz, CA, USA).

### Luciferase Reporter Assay

SK-N-DZ cells were co-transfected with MYCN promoter luciferase reporters (100 ng), kindly provided by Professor Akira Nakagawara of Chiba Cancer Center, Japan, and pRL-TK Renilla luciferase cDNA (10 ng) together with human AHR expression plasmid (pEGFP-C1-AHR) or pEGFP-C1 control vector (400 ng). Twenty-four hours after transfection, the firefly and Renilla luciferase activities were measured using the Dual-luciferase reporter assay system according to the manufacturer’s instructions (Promega).

### Statistical Analysis

The correlations between AHR and E2F1 or MYCN mRNA expression level were analyzed using the non-parametric Wilcoxon rank-sum test and Spearman’s correlation test. All the other quantitative real-time PCR data were analyzed for statistical significance by one-way analysis of variance followed by the Fisher’s protected least-significant difference test. The statistical analyses were performed using StatView software (Abacus Concept, Berkeley, CA, USA). Each result was obtained from at least three independent experiments and expressed as mean ± standard deviation. A p-value of p<0.05 was considered to be statistically significant for all tests.

## Results

### Microarray Analysis of Gene Expression Associated with MYCN

**Figure 1:**
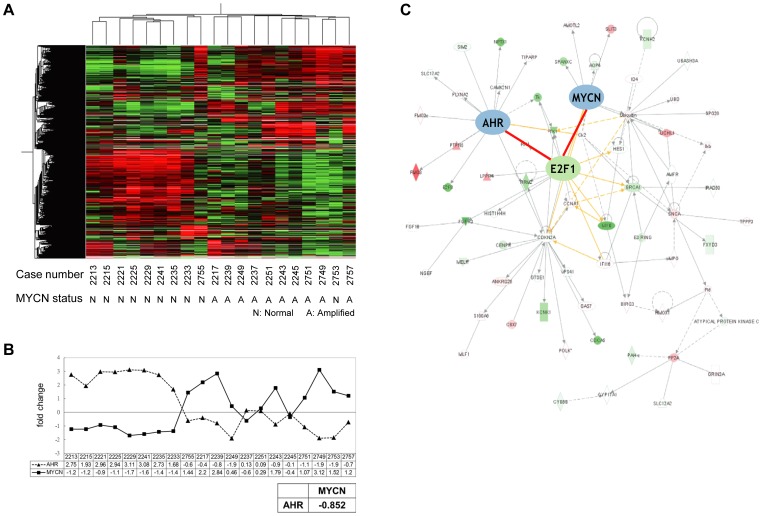
Oligonucleotide microarray analysis of NB tumors shows an inverse correlation between AHR and MYCN. (A) A dendrogram showing that 10 tumors with and 10 without MYCN amplification demonstrate two distinct gene expression patterns. (B) The inverse correlation between AHR and MYCN. The correlation coefficient was −0.852. (C) The differentially expressed genes in neuroblastoma were analyzed using the Ingenuity Pathway Analysis tool. The major functional networks showed that AHR and MYCN can interact through E2F1.

cDNA microarray has recently become a powerful tool to systemically evaluate differential gene expression [32]. To evaluate the expression of genes associated with MYCN in NB, 10 tumors with MYCN amplification and 10 with normal MYCN copy number were subjected to oligonucleotide microarray using Agilent oligo microarray chips (20173 genes). In the 10 patients with MYCN amplification, 6 patients died of disease at a follow-up of 11 to 57 months (median 17 months), whereas in the 10 patients with normal MYCN copy number, only 1 patient died of disease at a follow-up of 10 to 200 months (median 60 months). This data indicated that MYCN amplification is an extremely unfavorable prognostic factor of NB. A group of 2718 (13.5%) genes were selected that had abundance levels of at least 3 times the median abundance in at least 2 of the samples. In unsupervised analysis, there were two distinct gene expression patterns, which corresponded to cases with and without MYCN amplification (Fig. 1A). This result demonstrated that MYCN had a significant impact on the genome-wide NB gene expression.

### AHR Involvement in One of the Significant Functional Networks in NB

To further clarify the molecular regulation and gene expression profiles, the 2718 genes that were found to be differentially expressed in the unsupervised microarray analysis were analyzed using the IPA tool and were sorted into categories according to their annotation in IPA. The IPA knowledge-based database provides annotations regarding biological functions and signaling. Four significant genetic networks are ranked with the highest score, with molecular functions including (1) Cancer, Tumor Morphology, Cellular Growth and Proliferation; (2) Cell Cycle, Cellular Assembly and Organization, DNA Replication, Recombination, and Repair; (3) Neurological Disease, Dermatological Diseases and Conditions, Drug Metabolism; and (4) Lipid Metabolism, Small Molecule Biochemistry, Endocrine System Development and Function (Table 1). Among the genes involved in these four networks, AHR, which is the intracellular receptor for dioxin-like compounds, attracted our interest. Our array data showed the AHR expression was inversely correlated to MYCN expression in the 20 NB samples studied, with a correlation coefficient of −0.852 (Fig. 1B). Further functional protein-protein interaction analysis revealed that AHR could interact with MYCN through E2F1 (Fig. 1C).

**Table 1:** The first rank of significant genetic networks in differentially expressed genes in neuroblastoma.

Molecules in Networks	Score	FocusMolecules	Top Functions
AHR, ANKRD25, CAMK2N1, CBX7, CCNA1, CDCA5, CDKN2A, CENPK, E2F1, E2F8,FGF18, FGFR3, FMO2, FMO3, GAS7, GTSE1, HIST1H4H, HN1, KCNK1, LPPR4, MELK,MLF1, NGEF, NPTX1, PLXNA2, POLK, PTPN5, RRM2, S100A6, SIM2,SLC17A2, TIPARP, Tk, TK1, VPS41	36	34	Cancer, Tumor Morphology, Cellular Growth and Proliferation
AHNAK, BUB1, CDC45L, CDKN3, CENPA, CENPE, CIB2, CLEC4E, CRLF1, CX3CR1,EX01, IFIT2, IGJ, IL6, KIAA0101, KIF11, KIFC1, KLK8, MAD2L1,NDC80, NMU, NUF2, OIP5, PTGER3, RAG1, Rbp, RBP5, RBP7,SERPINA7, SNX10, SPC25, SV2B, TIMD4, TTR, ZWINT	36	34	Cell Cycle, Cellular Assembly and Organization, DNA Replication, Recombination and Repair
ALCAM, ANKRD15, ANXA6, ARG1, ARHGDIG, ATAD2, BBC3, CDCA7, COL5A1,COL5A2, EMP1, FAM129A, FBXO2, FXYD1, HSPH1, IGF2BP1, LIMA1, MAN1A1,MAN2A1, MAN2A2, Mannosidase Alpha, MAP4, MYC, NDRG1, OAS1, PAM, PERP, PHF5A,PLA1A, PMP22, PRKACB, PSAT1, RFX2, SHMT1, STMN1	36	34	Neurological Disease, Dermatological Diseases and Conditions, Drug Metabolism
3 BETA HSD, ADD3, AGT, AMH, BCL11A, BSN, CCL15, CTSG, CYP11A1, CYP11B2,ELA2, ERC1, F5, FDX1, FDXR, FGF9, GSTA3, HSD11B1, HSD3B1, HSD3B2, INA,KIF3A, LTF, NPPB, NPR3, NR0B1, NR2F1, NR2F2, RARRES2, RIMS2, SNF1LK, SPINK5, STAR, TNS1, ZFPM2	36	34	Lipid MetabolismSmall Molecule BiochemistryEndocrine System Development and Function

### AHR Expression was Inversely Correlated with MYCN in NB Tumors

**Figure 2:**
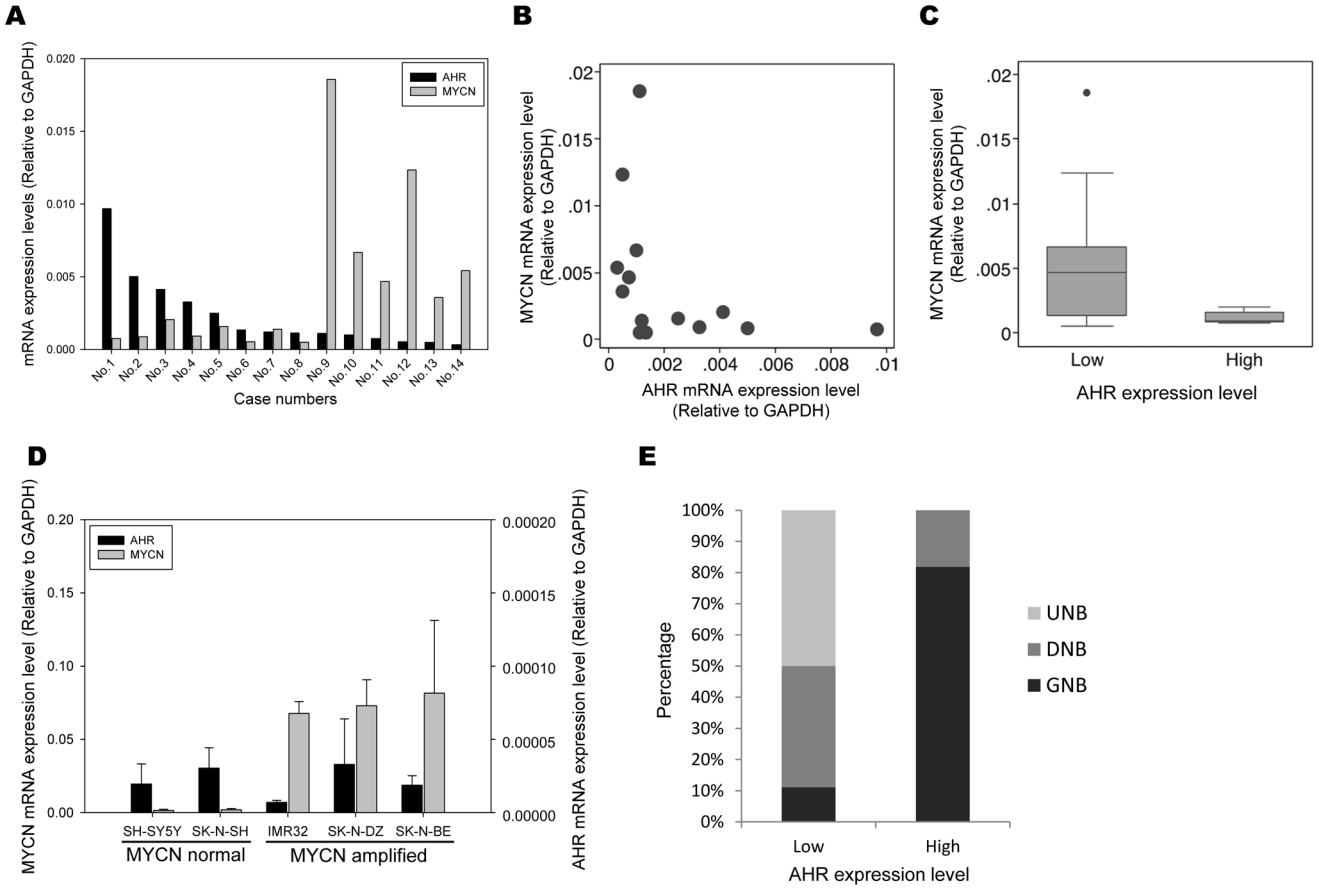
The correlation between AHR and MYCN mRNA expression level and differentiation histology in tumor samples of patients with NB. (A) Total mRNA of patient tumor samples were isolated by TRIzol reagent. The mRNA expression level of AHR and MYCN were analyzed using SYBR-green real-time PCR using gene specific primers. (B) The inverse correlation were analyzed by Spearman’s correlation test (Spearman’s ρ = −0.6484, p = 0.0121) (C) AHR expression level was analyzed in tumors with high and low MYCN expression status. (D) The total mRNA of MYCN non-amplified NB cell lines, SY5Y and SK-N-SH, and MYCN amplified NB cell lines, SK-N-BE, SK-N-DZ, and IMR32, were collected and subjected to SYBR-green real-time PCR with specific primers for AHR and MYCN. (E) The correlation between AHR expression level and differentiation of NB tumor histology was analyzed in 34 human NB tumor samples.

To further verify the correlation between AHR, E2F1, and MYCN, the mRNA expression levels of AHR and MYCN in another 14 NB tumor samples were determined by real-time PCR. The results showed a significantly negative correlation between AHR and MYCN (Fig. 2A and 2B, Spearman’s ρ = −0.6484, p = 0.0121). Using the non-parametric Wilcoxon rank-sum test, the MYCN expression level is significantly lower in tumors with higher AHR expression status (Fig. 2C, p = 0.0253). However, the correlation between AHR and E2F1 was not clear in this analysis (Fig. S1). There was a negative correlation between AHR and E2F1 expression levels (Fig. S1A and S1B, Spearman’s ρ = −0.4593, p = 0.0985) that was not statistically significant. E2F1 expression levels were higher in tumors with lower AHR expression status (Fig. S1C, p = 0.0476 by Wilcoxon rank-sum test).

The gene expression levels of AHR were also examined in five NB cell lines. The results showed that mRNA expression levels of AHR in all of the five NB cell lines were quite low, approximately 2×10^−5^-fold relative to the house-keeping gene GAPDH. Nevertheless, the AHR-to-MYCN expression ratio was still higher in NB cell lines with normal MYCN copy number than that in NB cell lines with MYCN amplification (Fig. 2D).

### AHR Expression was Correlated with the Differentiation of NB Tumor Histology

To further confirm the role of AHR in NB differentiation, the relationship between AHR and the differentiation status of NB tumors was analyzed. The histology of the 34 tumor samples of patients with NB included in this study were categorized based on the International Neuroblastoma Pathology Classification scheme [24]. Expression levels of AHR were analyzed using RT-PCR and real-time PCR. It was found that NB tumors with high levels of AHR expression had higher histological grades of differentiation (Fig. 2E), suggesting that AHR expression correlated to the differentiation of NB tumor tissues.

### AHR Overexpression Downregulates MYCN Expression and Promotes Neural Differentiation of NB Cells

**Figure 3:**
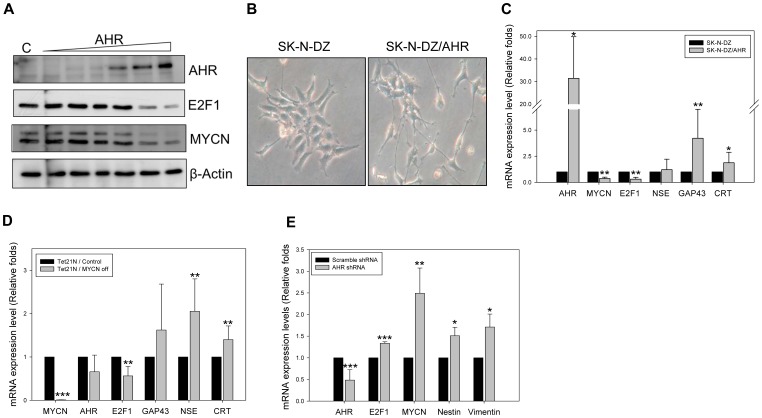
AHR regulates MYCN expression level in NB cell lines. (A) SK-N-SH cells were transfected with increasing amounts of AHR by a lentivirus transduction system. The cell lysate was collected by lysis buffer with proteinase inhibitors and analyzed by Western Blot. (B) The SK-N-DZ cell line was transfected with human AHR expression vector (pEGFP-C1-hAHR) by Lipofectamine 2000 and selected by G418 antibiotics. By morphology observation, AHR overexpression promotes neurite outgrowth of SK-N-DZ cell line. (C) 1 µg of total RNA, isolated by TRIzol reagent from SK-N-DZ/AHR cells, was reverse-transcribed to cDNA. The expression levels of each gene were quantified by SYBR-Green real-time PCR. AHR overexpression in SK-N-DZ cells suppressed E2F1 and MYCN expression and upregulated the mRNA expression level of differentiation markers, GAP43 and CRT. (D) Tet21N is a neuroblastoma cell line with tetracycline-controlled expression of MYCN (Tet-off). 10 ng/ml tetracycline was added to the growth media for 24 h to conditionally knockdown MYCN expression. After tetracycline treatment, the total mRNA were collected and subjected to real-time PCR analysis with gene specific primers. (E) SK-N-SH cells were transfected with AHR shRNA by lentivirus transduction system. After 48 h, total mRNA were collected and subjected to real-time PCR with specific primers for AHR, E2F1, MYCN, Nestin, and Vimentin.

The SK-N-SH NB cell line was transiently transfected with increasing amounts of AHR expression plasmid using the lentivirus transduction system. The immunoblotting data showed that ectopic expression of AHR downregulated E2F1 and MYCN expression in a dose-dependent manner (Fig. 3A). Furthermore, we attempted to establish a stable AHR overexpressing NB cell line. SK-N-DZ, a MYCN-amplified NB cell line, was transfected with human AHR expression plasmid (pEGFP-C1-AHR) using Lipofectamine 2000. After 1 month of G418 selection, spontaneous neurite outgrowth was observed in drug resistant SK-N-DZ/AHR cells, which might indicate neural differentiation (Fig. 3B). CYBR-green real-time PCR was then performed to detect AHR mRNA expression. Over 30 folds of AHR expression were detected in the SK-N-DZ/AHR cells as compared with wild type SK-N-DZ cells (Fig. 3C). In addition, MYCN and E2F1 mRNA expression levels were downregulated in SK-N-DZ/AHR cells (Fig. 3C). Furthermore, the expression levels of neuron-specific markers, including growth-associated protein-43 (GAP-43), neuron-specific enolase **(**NSE), and calreticulin (CRT) were also upregulated in SK-N-DZ/AHR cells (Fig. 3C). Taken together, our results suggest that the AHR overexpression downregulates MYCN expression and promotes neural differentiation in NB.

### AHR is One of the Upstream Regulators of MYCN

To further clarify the regulatory relationship between AHR and MYCN in NB, AHR expression levels were detected using real-time PCR in Tet21N cells, a cell line containing a tetracycline-regulated MYCN transgene (Tet off). In this cell system, tetracycline treatments efficiently suppressed MYCN expression (Fig. 3D). Our results show that the neural differentiation markers NSE and CRT were both upregulated in tetracycline treated Tet21N cells. However, there was no significant change in AHR expression level when MYCN was conditionally downregulated. This result suggests that AHR might be an upstream regulator of MYCN.

Next, we tried to use AHR shRNA to determine whether downregulation of AHR affects MYCN expression in NB cells. Using a lentivirus transduction system, shRNA efficiently suppressed AHR expression in SK-N-SH cells with an upregulation of E2F1 and MYCN mRNA expression (Fig. 3E). A significant upregulation of the undifferentiated neuron-markers Nestin and Vimentin revealed a blockage in neuronal differentiation in AHR knockdown SK-N-SH cells (Fig. 3E). These results confirm our hypothesis that AHR is possibly one of the upstream regulators of MYCN.

### AHR Downregulated MYCN Expression through Regulating MYCN Promoter Activity

**Figure 4:**
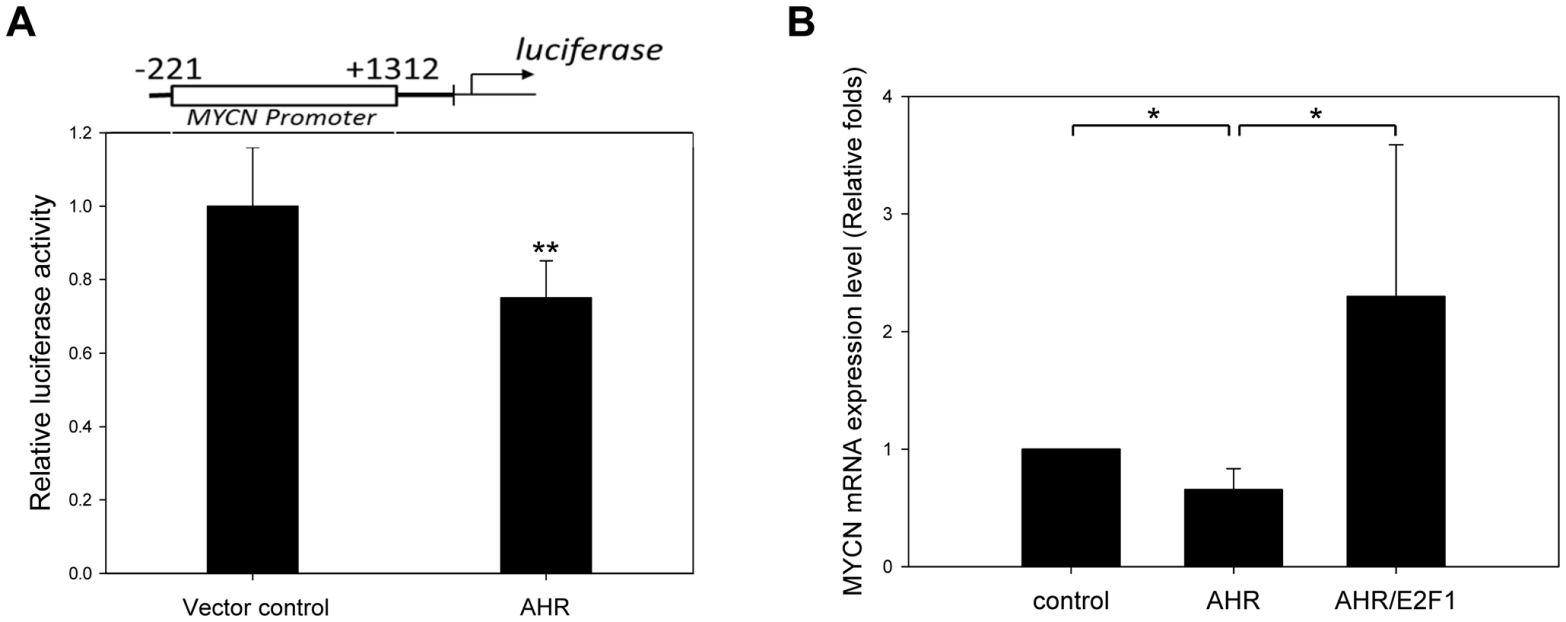
AHR regulates MYCN gene expression through the modulation of E2F1 and MYCN promoter activity. (A) SK-N-DZ cells were transiently co-transfected with a constant amount of MYCN promoter luciferase reporter plasmid (100 ng) and Renilla luciferase reporter plasmid (pRL-TK) (10 ng) along with human AHR expression plasmid (pEGFP-C1-hAHR) or pEGFP-C1 (400 ng). Twenty-four hours after transfection, cells were lysed and luciferase activities were measured. Firefly luminescence signal was standardized by the Renilla luminescence signal. (B) SK-N-SH cells were co-transfected with human AHR expression plasmid by a lentivirus transduction system along with E2F1 expression plasmid or control vector (pLKO_AS2). After 48 h, total mRNA were collected and subjected to RT-PCR. MYCN mRNA was analyzed by SYBR-Green real-time PCR.

To examine whether AHR suppresses MYCN gene expression through regulation of its promoter, MYCN promoter activity was analyzed by luciferase reporter assay after AHR overexpression. SK-N-DZ cells were transiently co-transfected with MYCN promoter luciferase reporter plasmid and Renilla luciferase reporter plasmid (pRL-TK) along with human AHR expression plasmid (pEGFP-C1-hAHR) or a control plasmid (pEGFP-C1). The quantitative results showed that the MYCN-promoter-derived luciferase activity was downregulated by AHR overexpression (Fig. 4A). This result indicates that AHR could downregulate MYCN expression through the suppression of MYCN promoter activity.

### E2F1 is Involved in the Pathway of AHR Regulating MYCN Gene Expression

Using IPA, we also found that AHR could interact with MYCN through E2F1. Therefore, we hypothesized that AHR might downregulate MYCN expression through modulation of E2F1. In this study, we found that the protein and mRNA expression level of E2F1 were downregulated by AHR overexpression in NB cells (Fig. 3A and 3C). Conversely, knockdown AHR promoted E2F1 mRNA expression (Fig. 3E). To further confirm that E2F1 is involved in the pathway of AHR regulating MYCN gene expression, we overexpressed E2F1 in AHR overexpressing SK-N-SH cells. The effect of downregulation of MYCN mRNA by ectopic expression of AHR was compensated for by E2F1 overexpression (Fig. 4B). However, the direct interaction of AHR and E2F1 or MYCN was not observed in immunoprecipitation experiments (Fig. S2) and immunocytochemistry staining (Fig. S3) in our NB cell system.

## Discussion

Our results show that the molecular markers of NB have a significant impact on tumor behavior [33]. MYCN amplification is the most well-characterized marker that predicts a poor outcome in patients with NB. However, it is still unclear how MYCN affects NB cell behavior. In this study, we employed microarray and bioinformatics tools (in IPA analysis) to search for candidate genes involved in the regulation of MYCN expression in NB. The unsupervised analysis of microarrays showed two distinct gene expression patterns, which corresponded to cases with and without MYCN amplification (Fig. 1A). This result confirmed that MYCN has a significant impact on the genome-wide NB gene expression and may affect NB cell behavior. Using oligonucleotide microarrays, 2718 differentially expressed NB genes were identified. Among these genes, AHR was found to be inversely correlated to MYCN amplification and had the highest score in IPA analysis.

Recent studies have suggested that AHR plays an important role in neurogenesis and neural differentiation. 2,3,7,8-tetrachlorodibenzo-p-dioxin (TCDD) disrupts the normal physiological activity of AHR, resulting in compromised granule neuron precursor cell differentiation [34]. AHR-deficient mice display diminished neuronal differentiation in the dentate gyrus [35]. A recent study of ours also revealed that the silencing of miR-124 induces SK-N-SH cell differentiation by promoting AHR [36]. In the present study, we found that the AHR expression is inversely correlated with MYCN in NB tumors using microarray analysis. This relationship was further confirmed by real-time PCR in both NB tumors and NB cell lines. Furthermore, the expression level of AHR was found to be correlated with the histological grade of differentiation in NB tumors. Knockdown of MYCN has been reported to promote neural differentiation in NB cells [37], [38]. Our results show that AHR overexpression might suppress MYCN expression and that NB cells with AHR overexpression eventually differentiate in a ligand-independent manner. Thus, AHR in NB is likely to act as a tumor suppressor and promote tumor differentiation by downregulation of MYCN.

Dioxin is known to be the ligand of AHR. In humans, long term epidemiological studies have suggested that there is a strong link between high dioxin exposures and certain types of cancer and cardiovascular diseases [39]–[42]. Moreover, it has been reported that exposure of pregnant rats to dioxin reduces AHR expression levels in the fetus [43]. In addition, it has been reported that children of parents with dioxin exposure may be at increased risk of NB [44]. In this study, we demonstrated an inverse relationship between AHR and MYCN expression both *in vitro* and *in vivo*. Taken together, it is plausible that parents with dioxin exposure might cause AHR suppression in their children, which in turn might lead to MYCN overexpression and the occurrence of NB. This hypothesis suggests a possible molecular basis for the role of environmental pollutants, such as dioxin, in the pathogenesis of NB.

E2F1 is a transcription factor that is crucial for the transition of the cell cycle from the G1 phase to the S phase [45]. Its activity is negatively controlled by the pRb pathway [46]. E2F1 activation turns on the expression of a variety of genes required for DNA replication and promotes cell proliferation [47], [48]. Recent studies have suggested that AHR might regulate E2F1 activity. E2F1-dependent transcription and cell cycle progression have been shown to be suppressed by interaction with AHR [19]. AHR may bind to E2F1 and inhibit E2F1-induced apoptosis [17]. AHR may also regulate cell proliferation via transcriptional activation of E2F1 in A549 cells [20]. Consequently, AHR may act as a coactivator/suppressor of the E2F1 transcription factor, in addition to its role as a transcription factor. Furthermore, E2F1 has been shown to be critical for both the full activation and repression of MYCN in NB [49]. Our IPA analysis suggests that AHR may interact with MYCN via E2F1. AHR overexpression in SK-N-DZ and SK-N-SH NB cell lines downregulated the expression of E2F1 and MYCN. Moreover, the expression levels of E2F1 and MYCN were upregulated by AHR knockdown. These lines of evidences suggested that AHR in NB may serve as a suppressor of E2F1 to downregulate MYCN expression. It has been shown that AHR could directly interact with and modulate the transcriptional activity of E2F1 [17], [18], [20]. Moreover, a recent study demonstrated that E2F1 can bind to the MYCN promoter and regulate MYCN expression in NB [49]. Our results further indicate that AHR could downregulate MYCN expression through suppression of MYCN promoter activity. However, the direct interaction between AHR and E2F1 or MYCN was not observed in our NB cell system. Further studies are required to elucidate the specific mechanism of how AHR interacts with E2F1 to regulate MYCN promoter activity.

Here, we show that by microarray and IPA analysis in NB tumor samples, the regulation of MYCN expression is closely related to AHR expression. There was a strong inverse correlation between AHR expression in MYCN in both NB tumors and cells. Ectopic expression of AHR in NB cells may not only suppress MYCN expression but also promote spontaneous cell differentiation. Downregulation of AHR by pollutants such as dioxin in embryonic neuroblastic cells could possibly lead to upregulation of MYCN and hence the occurrence of NB tumors. Further investigation of the regulatory role of AHR in the expression of MYCN may shed light, not only on the pathogenesis of NB, but also on a novel prevention or treatment for this devastating cancer.

## Supporting Information

Figure S1 **The correlation between AHR and E2F1 mRNA expression level in tumor samples of patients with NB.** (A) Total mRNA of patient tumor samples were isolated by TRIzol reagent. The mRNA expression level of AHR and E2F1 were analyzed using SYBR-green real-time PCR with gene specific primers. (B) The inverse correlation was analyzed by Spearman’s correlation test (Spearman’s ρ = −0.4593, *P* = 0.0985) (C) AHR expression level was analyzed in tumors with high and low MYCN expression status.
(TIFF)

Figure S2 **Co-immunoprecipitation of AHR and E2F1 in SK-N-DZ cells.** SK-N-DZ cells were overexpressed AHR-His by lenti-viral transduction system. Total cell lysates were immunoprecipitated with mouse anti-AHR and mouse anti-His antibodies. Protein A/G-bound antigen-antibody complexes were analyzed by immunoblotting with anti-AHR (upper panel) and anti-E2F1 (lower panel) antibodies.
(TIF)

Figure S3 **Double immunofluorescence staining of AHR and E2F1 or MYCN in SK-N-DZ cells.** SK-N-DZ cells were transfected with human AHR expression plasmid for 48 hr and processed for immunofluorescence staining with goat anti-AHR (green), rabbit anti-E2F1 (red) and mouse anti-MYCN (red) antibodies. Nuclei were counterstained with DAPI (blue).
(TIF)
