# Correction: Aryl Hydrocarbon Receptor Downregulates MYCN Expression and Promotes Cell Differentiation of Neuroblastoma

**DOI:** 10.1371/journal.pone.0100419

**Published:** 2014-06-11

**Authors:** The PLOS ONE Staff

The sixth author’s name is spelled incorrectly. The correct name is: Yen-Lin Liu.
